# Athlete Medical Services at the Marathon and Race Walking Events During Tokyo 2020 Olympics

**DOI:** 10.3389/fspor.2022.872475

**Published:** 2022-04-22

**Authors:** Makoto Sugawara, Yoshiaki Manabe, Fumihiro Yamasawa, Yuri Hosokawa

**Affiliations:** ^1^Medical Committee, Japan Association of Athletics Federations (JAAF), Tokyo, Japan; ^2^Matsuda Orthopedic Memorial Hospital, Sapporo, Japan; ^3^Department of Sports Science, Chukyo University, Nagoya, Japan; ^4^Marubeni Health Promotion Center, Tokyo, Japan; ^5^Faculty of Sport Sciences, Waseda University, Saitama, Japan

**Keywords:** Olympic Games, marathon, race walk, exertional heat illness, race medicine

## Abstract

Epidemiological data from race walk and marathon events suggest that a high incidence rate of exertional heat illness is associated with high ambient temperature and relative humidity. The 2020 Summer Olympics in Tokyo was no exception, which led the organizing committee to relocate the race walk and marathon competitions to Sapporo, which was predicted to experience much milder heat. Nonetheless, during the Games, Sapporo recorded the highest daytime ambient temperature in the past 97 years, with consecutive days over 30°C from July 22nd to August 7th, 2021. Five events (men's and women's 20 km race walk, men's 50 km race walk, women's and men's marathon) were held in Sapporo from August 5th to August 8th, 2021. The percentage of athletes who did not finish (DNF) in each event was 8.8% in men's 20 km race walk, 20.3% in men's 50 km race walk, 8.6% in women's 20 km race walk, 17.1% in women's marathon and 28.3% in men's marathon. A total of fifty athletes were transferred to the athlete medical station: 28 athletes completed the race (i.e., collapsed after finish line), while 24 were DNF athletes transported from the course. Forty-eight (96%) of athletes who were admitted to the athlete medical station exhibited signs and symptoms of exertional heat illness. Two athletes diagnosed with exertional heat stroke and three athletes diagnosed with severe heat exhaustion (rectal body temperature >39.5°C with or without central nervous system disturbance) were cooled using whole-body cold water immersion at the heat deck located within the athlete medical station. All athletes who were cooled successfully recovered without any complications. These athletes required an average of 14 ± 9.4 min (range, 6–30 min) to cool their rectal temperature below 39°C. These results show the importance for event organizers to prepare strategies to keep athletes cool, such as an ample amount of ice and water to supply whole-body cold water immersion.

## Introduction

The 2020 Summer Olympics in Tokyo, which was held from July 24th to August 9th, 2021, was anticipated to be characterized by extreme heat conditions, and athletes were projected to experience environmental heat stress that surpasses the environmental conditions observed in previous Summer Olympics (Kakamu et al., 2017). These challenging environmental conditions led the Athlete365, an International Olympic Committee (IOC) initiative created by athletes, for athletes, whose mission is to support athletes' journey in sport on and off the field, and the IOC Medical Department to promote educational materials that covered ways for athletes to prepare for the heat well-ahead of time (Athlete365 Beat the heat at Tokyo, 2020). Exertional heat illness (EHI) is one of the leading medical concerns associated with competitions that are scheduled under high ambient temperature (T_a_) and high relative humidity (RH). EHI is further classified into the following conditions by its etiology: exercise-associated muscle cramps (EAMC), heat syncope (HS), heat exhaustion (HE), and exertional heat stroke (EHS) (Armstrong et al., 2007; Hosokawa et al., 2019). In particular, EHS, the most severe form of EHI, is regarded as a medical emergency that could be fatal if not immediately diagnosed and promptly properly treated. As the environmental heat risk was expected to be oppressive in Tokyo, the Tokyo Organizing Committee of the Olympic and Paralympic Games had decided to relocate the Olympic race walk and marathon competitions to Sapporo, which is ~800 km north of Tokyo, whose historical environmental condition (2010–2020) shows much milder average high T_a_ and equivalent average RH (27.1°C, 74.7%) to Tokyo (average high T_a_, 30°C; average RH, 74.8%) in August (Japan Meteorological Agency Past Weather Database). Nevertheless, Sapporo recorded the highest T_a_ in the past 97 years, with consecutive maximal daytime T_a_ exceeding 30°C from July 22nd to August 7th, 2021. While the venue in Sapporo was equipped with resources to perform standardized EHS prehospital management (Hosokawa et al., 2021), the athlete medical services at Sapporo urgently implemented additional countermeasures against EHI risk. For example, the decision to move the start time of the women's marathon by 1 h, from 7 am to 6 am, was made 1 day before the race, given the unexpectedly hot environmental condition leading up to the day of the race.

While EHI is not a new concern for Summer Olympic Games, no publications to date have reported the epidemiology of EHI during the Summer Olympic Games. As we are projected to face more extreme heat events in the future (Ebi et al., 2021), unexpected heat waves may be observed irrespective of the historical climate patterns. Therefore, we first aimed to describe the pattern of EHI sustained by race walk and marathon athletes in Sapporo, Japan, during the 2020 Summer Olympic Games in Tokyo. Second, we aimed to summarize the outcome of athletes who were treated using the standardized EHS prehospital management in the Olympic athlete medical services. Findings from this article will help the future elite endurance event organizers gauge the extent of medical resources and services that must be prepared to optimize athletes' safety.

## Methods and Materials

### Event Settings

Five events (men's and women's 20 km race walk, men's 50 km race walk, women's marathon, and men's marathon) were held at Sapporo from August 5th to August 8th, 2021 (Table 1). Men's 50 km race walk, men's marathon, and women's marathon were scheduled in the early morning to minimize the influence of solar radiation and ambient temperature. The race walk used a loop course starting at Odori Park, using 20-laps of 1 km circuit course for the 20 km race walk, and 25-laps of 2 km circuit course for the 50 km race walk. The start and the end of the Marathon course were also set at Odori Park. The marathon course consisted of a large loop, which covered the first 20 km of the race, followed by a smaller loop, ~10 km in the distance, which the athletes ran twice, totaling three laps. All athletes who required medical attention were transferred to the athlete medical station, which was located adjacent to the finish line.

**Table 1:** Event schedule.

**Date**	**Event**	**Start time**	**Participants**
August 5th, 2021	Men's 20 km race walk	16:30	57
August 6th, 2021	Men's 50 km race walk	5:30	59
August 6th, 2021	Women's 20 km race walk	16:30	58
August 7th, 2021	Women's marathon	6:00^a^	88
August 8th, 2021	Men's marathon	7:00	106

a *Originally scheduled to start from 7:00 but the start time was moved 1-h earlier to avoid heat*.

### Exertional Heat Illness (EHI) Recognition and Treatment

The athlete medical station was equipped with rectal thermometers and ice baths to provide cold-water immersion when the collapsed athlete was diagnosed with exertional heat stroke (Hosokawa et al., 2021). Once the collapsed athlete was admitted to the athlete medical station, medical volunteers who were trained in the exertional heat stroke prehospital care determined the need to assess the athlete's internal body temperature using a rectal thermometer (DataTherm II Continuous Temperature Monitor, Geratherm Medical AG). Athletes with confirmed hyperthermia (rectal temperature ≥40.5°C) and central nervous system dysfunction were cooled using whole-body cold water immersion. Water temperature in the ice bath was kept between 10 and 15°C. Rectal temperature was monitored throughout the duration of cooling, and it was ceased when the rectal temperature reached <39°C. EHS diagnosis and treatment were carried out by a physician, nurses, and physical therapists.

### Medical Record Keeping

Medical volunteers stationed at the athlete medical station took medical records (e.g., physician, nurse, physical therapist). The data were first recorded on the paper chart and later entered into an electronic system for statistical analysis.

## Results

### Environmental Conditions

**Figure 1:**
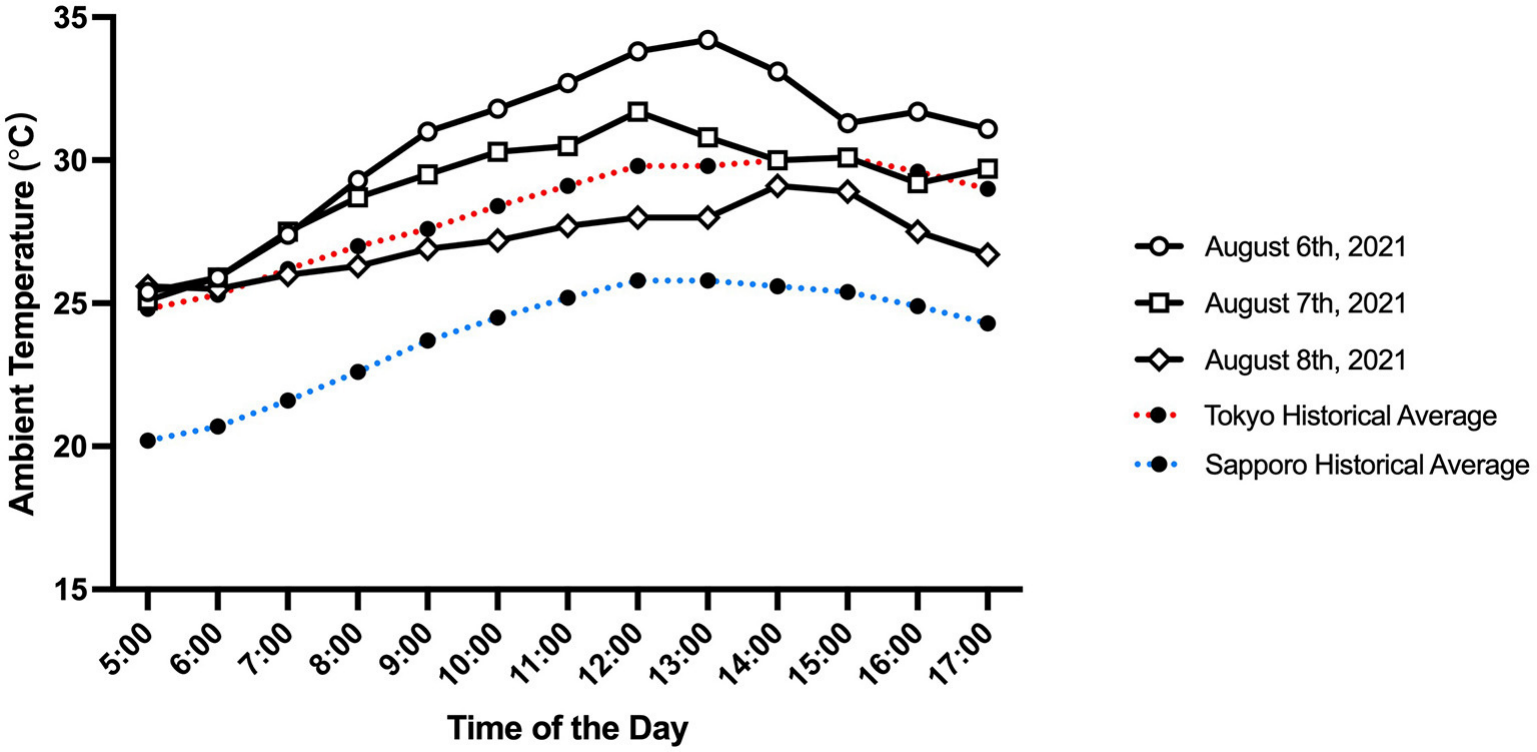
Ambient temperature observed in Sapporo on August 6th (men's 50 km race walk), 7th (women's marathon), and 8th (men's marathon), 2021, in Sapporo between 5:00 am and 5:00 pm. The red and blue dotted lines represent average historical ambient temperature (from July 21st to 31st, 2009 through 2018) in Tokyo and Sapporo, respectively.

**Figure 2:**
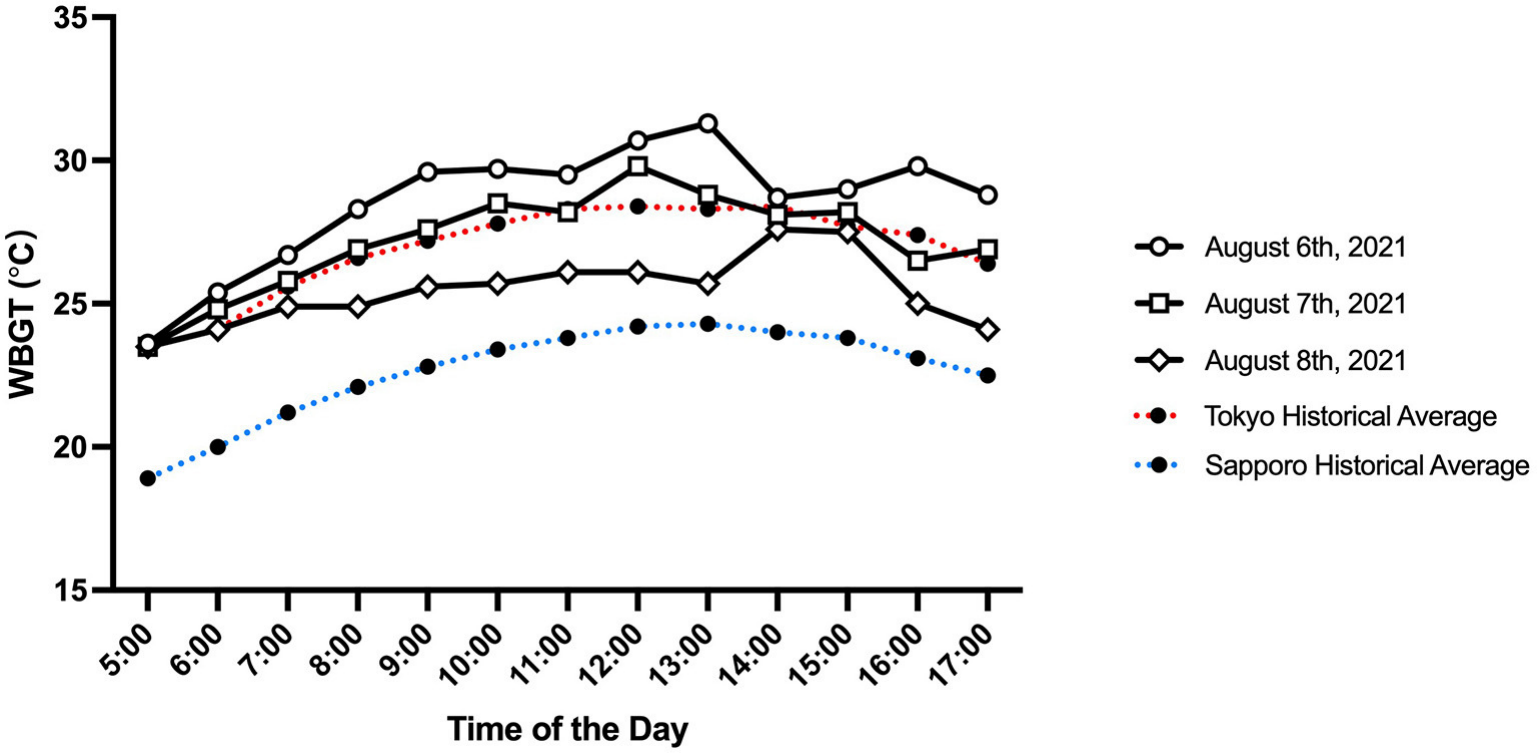
Wet bulb globe temperature observed in Sapporo on August 6th (men's 50 km race walk), 7th (women's marathon), and 8th (men's marathon), 2021, in Sapporo between 5:00 am and 5:00 pm. The red and blue dotted lines represent average historical ambient temperature (from July 21st to 31st, 2009 through 2018) in Tokyo and Sapporo, respectively.

Environmental conditions observed at the start line during men's 50 km race walk, women's marathon, and men's marathon are summarized in Figures 1, 2. The start time T_a_, RH, and wet bulb globe temperature (WBGT) suggest that the corresponding flag system for thermal stress as defined by World Athletics was “Caution (WBGT, 21 to 25°C)” (men's 50 km race walk, T_a_ = 25°C, RH = 86%, WBGT = 24°C; women's marathon, T_a_ = 25°C, RH = 84%, WBGT = 23°C; men's marathon, T_a_ = 28°C, RH = 72%, WBGT = 24°C) (Adami et al., 2020).

### Did Not Finish (DNF) Athletes

The percentage of DNF athletes in each event was 8.8, 8.6, 20.3, 17.0, and 28.3% in men's and women's 20 km race walk, men's 50 km race walk, women's and men's marathon, respectively (Table 2).

**Table 2:** Percentage of athletes who did not finish (%DNF).

**Event**	**Number of finishers**	**Number of DNF**	**%DNF**	**Completion rate**
Men's 20 km race walk	52	5	8.8%	91.2%
Women's 20 km race walk	53	5	8.6%	91.4%
Men's 50 km race walk	47	12	20.3%	79.7%
Men's marathon	76	30	28.3%	71.7%
Women's Marathon	73	15	17.0%	83.0%

*DNF, did not finish*.

### EHI Incidence Rates and Cooling Rates

A total of fifty athletes were transferred to the athlete medical station: 28 athletes completed the race (i.e., collapsed after finish line), while 22 were DNF athletes transported from the field of play.

Forty-eight (96%) of athletes who were admitted to the athlete medical station exhibited signs and symptoms of EHI. HE was most common (*n* = 22), followed by EAMC (*n* = 12), HS (*n* = 12), and EHS (*n* = 2) (Tables 3A,B).

**Table 3A:** Athletes treated at athlete medical service in men's and women's 20 km race walk and men's 50 km race walk.

**Event**		**Result**	**Diagnosis**	**Duration at AMS (minutes)**	**Method of cooling**	**Rectal temperature (**°**C)**
Men's 20 km race walk	1	DNF	EAMC	10	Ice towel, cold drink	–
	2	Finisher	EAMC	6	Ice towel	–
	3	Finisher	HE	19	Ice towel, cold drink	–
Women's 20 km race walk	4	DNF	HE	33	Ice towel	–
	5	DNF	HE	23	Ice towel, cold drink	–
	6	DNF	HE	20	Ice towel, cold drink	–
	7	Finisher	HS	8	Ice towel	–
Men's 50 km race walk	8	DNF	Knee pain	13	Ice towel, cold drink	–
	9	DNF	HS	5	Cold drink	–
	10	DNF	EAMC	15	Ice towel,	–
	11	DNF	EHS	60	CWI	42.65
	12	Finisher	EAMC	44	Ice towel, cold drink	–
	13	Finisher	HS	3	Ice towel	–
	14	Finisher	HS	16	Ice towel	–
	15	Finisher	EAMC	20	Ice towel	–
	16	Finisher	EAMC	8	Ice towel	–
	17	Finisher	HS	8	Ice towel	–
	18	Finisher	EAMC	18	Ice towel, cold drink	–
	19	Finisher	EAMC	39	Ice towel, cold drink	–

*AMS, athlete medical service; DNF, did not finish; EAMC, exercise associated muscle cramp; HE, heat exhaustion; HS, heat syncope; CWI, cold water immersion*.

**Table 3B:** Athletes treated at athlete medical service in men's and women's marathon.

**Event**		**Result**	**Diagnosis**	**Duration at AMS (minutes)**	**Method of cooling**	**Rectal temperature (**°**C)**
Men's marathon	20	DNF	HE	20	Cold drink	–
	21	DNF	EAMC	34	None	–
	22	DNF	HE	47	Ice towel, cold drink	–
	23	DNF	Skin wound	26	–	–
	24	Finisher	HE	14	Ice towel	–
	25	Finisher	Severe HE	31	CWI	40.01
	26	Finisher	HS	3	Ice towel	–
	27	Finisher	HS	16	Cold drink	–
	28	Finisher	HS	8	Ice towel, cold drink	–
	29	Finisher	EAMC	25	Ice towel	–
	30	Finisher	EHS	34	CWI	40.63
	31	Finisher	HE	15	Cold drink	–
	32	Finisher	HS	6	Ice towel	–
Women's marathon	33	DNF	HE	28	CWI, ice towel	–
	34	DNF	HS	10	Cold drink	–
	35	DNF	HS	5	Cold drink	–
	36	DNF	HE	19	Cold drink, rewarm	–
	37	DNF	HE	14	–	–
	38	DNF	HE	11	Ice towel	–
	39	DNF	EAMC	25	Ice towel, cold drink	–
	40	DNF	HE	–	–	–
	41	DNF	HE	27	Ice towel, cold drink	–
	42	DNF	HE	16	Ice towel	–
	43	Finisher	HE	13	Ice towel	38.94
	44	Finisher	HS	5	Ice towel	–
	45	Finisher	Severe HE	27	CWI	40.20
	46	Finisher	HE	15	Ice towel	–
	47	Finisher	HE	84	Ice towel, IV therapy	38.65
	48	Finisher	Severe HE	15	CWI	39.55
	49	Finisher	EAMC	10	Ice towel	–
	50	Finisher	HE	–	Ice towel	–

*AMS, athlete medical service; DNF, did not finish; EAMC, exercise associated muscle cramp; EHS, exertional heat stroke; HE, heat exhaustion; HS, heat syncope; CWI, cold water immersion; IV, intravenous*.

**Figure 3:**
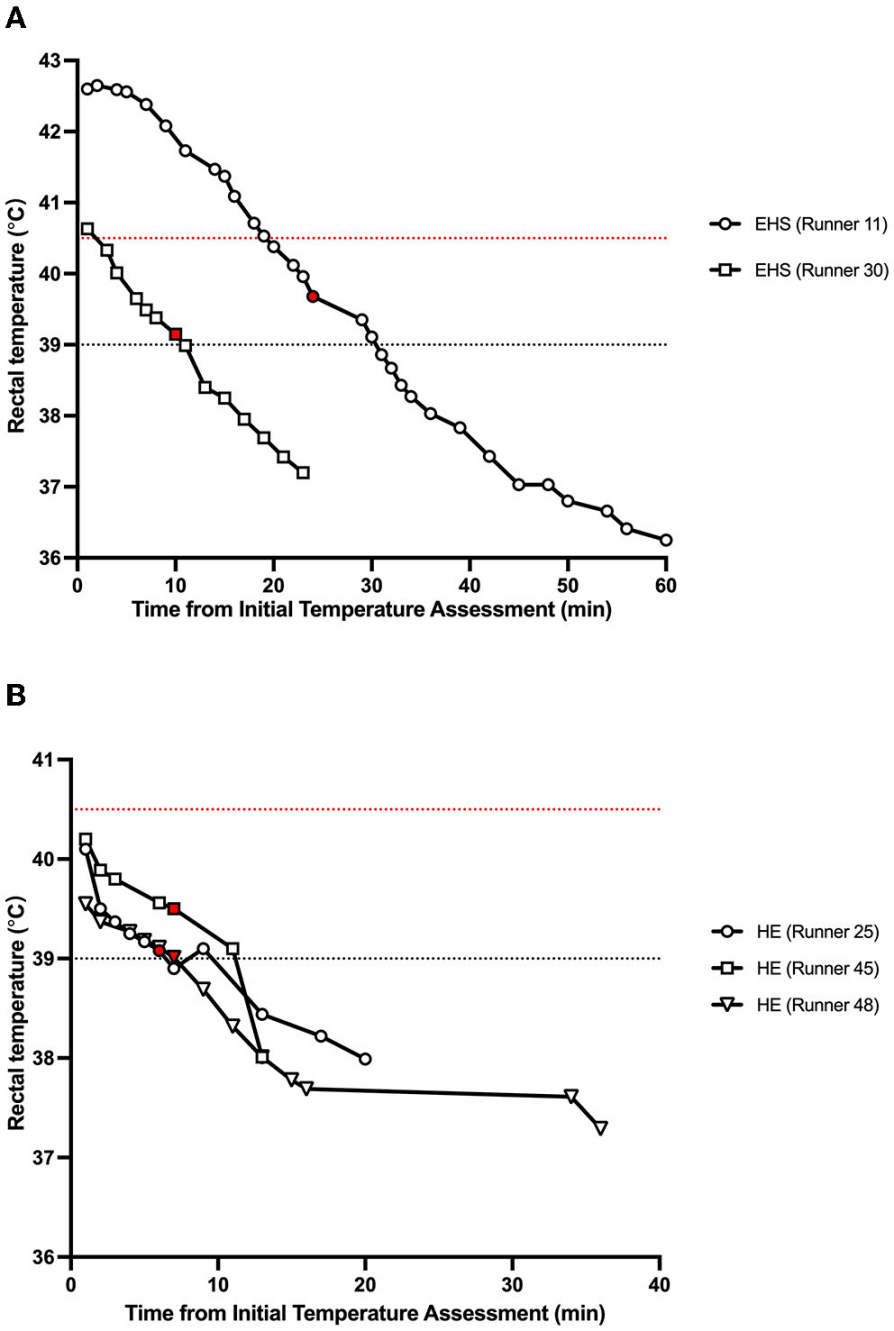
Summary of change in rectal temperature during and post whole-body cold water immersion of **(A)** exertional heat stroke and **(B)** severe heat exhaustion runners. Plots colored in red indicate the end of whole-body cold water immersion. Runner ID corresponds to Tables 3A,B. The red dotted line shows the reference value for exertional heat stroke diagnosis (40.5°C). The black dotted line shows the reference value for ending whole-body cooling (39.0°C).

EHI incidence rate per 1,000 finishers was highest in 50 km men's race walk (170.2 per 1,000 finishers), followed by men's marathon (118.4 per 1,000 finishers), women's marathon (109.6 per 1,000 finishers), men's 20 km race walk (38.5 per 1,000 finishers) and women's 20 km race walk (18.9 per 1,000 finishers) (Table 4). Two athletes diagnosed with EHS and three athletes with severe HE (rectal body temperature >39.5°C with or without central nervous system disturbance) were cooled using whole-body cold water immersion at the heat deck located within the athlete medical station. All athletes were cooled successfully and recovered without any complications. The average recorded cooling rate to reach 39°C was 0.11 ± 0.03°C (EHS, 0.13 ± 0.02°C; HE, 0.10 ± 0.02°C). These athletes required an average of 14 ± 9.4 min (range, 6–30 min) to cool their rectal temperature below 39°C (Figure 3).

**Table 4:** Exertional heat illness incidence rate per 1,000 finishers by event.

**Event**	**Number of finishers**	**Number of EHI in finishers**	**EHI per 1,000 finishers**
Men's 20 km race walk	52	2	38.5
Women's 20 km race walk	53	1	18.9
Men's 50 km race walk	47	8	170.2
Men's marathon	76	9	118.4
Women's marathon	73	8	109.6

*EHI, exertional heat illness*.

## Discussion

### Environmental Conditions

Epidemiological data from race walk and marathon events suggest that a high incidence rate of EHI is associated with high T_a_ and RH (Armstrong et al., 2007; Hosokawa et al., 2018). The 2020 Summer Olympics in Tokyo was no exception, which led the Tokyo Organizing Committee of the Olympic and Paralympic Games to relocate the race walk and marathon competitions to Sapporo, which was predicted to experience much milder heat. A study conducted after the announcement of the relocation revealed that Sapporo was indeed modeled to experience less thermal stress than Tokyo when the Physiologically Equivalent Temperature and the modified Physiologically Equivalent Temperature were calculated (Wu et al., 2020). Another study conducted at a similar time examined the predicted WBGT and universal thermal climate index (UTCI) between Tokyo and Sapporo during the Games (Seo and Honjo, 2021). Their study also suggested a milder heat strain in Sapporo than in Tokyo, where the predicted average WGBT and UTCI were lower by 4.4 and 6.3°C, respectively. Nonetheless, Sapporo experienced extreme heat during the Games (Figures 1, 2), which suggests that a data-driven preparation could not sufficiently detect the risk of extreme heat. In fact, the %DNF and EHI incidence rates in women's marathon could have been much worse if the competition had started at the scheduled time since the WBGT was already at 27.5°C by 7:00 am. Therefore, future races should consider a much earlier start, such as before sunrise (i.e., 4:00 am), which would allow the race to conclude around 6–7 am. In addition, future organizers of elite endurance events should prepare a contingency plan for unexpected heat events to make sure that the event operation has the flexibility to accommodate sudden changes in the event schedule. The contingency plan should, at minimum, address arrangements of event and medical volunteers, traffic and road closure controls, audience control and security, ambulance services, and broadcasting and media relations.

### DNF Athletes

The percentage of DNF athletes documented at Sapporo was less in all events except for the men's marathon when compared to the World Athletics Championships in Doha, 2019. They experienced one of the most extreme heat events for elite endurance competitions in recent years (event start T_a_ range, 29–32°C; event start RH range, 51–77%) (Bermon and Adami, 2019), resulting in %DNF of 23.1, 13.3, 39.1, 24.7, and 41.2% in men's and women's 20 km race walk, men's 50 km race walk, women's and men's marathon, respectively. The reduction in %DNF under comparable environments may have been due to increased preparedness among athletes since the Doha World Championships.

### EHI Incidence Rates

Almost all (96%) of the athletes who were transferred to athlete medical station were EHI. Five athletes required cold water immersion due to EHS and severe HE. EHI incidence rates at the Olympic race walks and marathons in this study were high compared to other mass-participation endurance events. For example, the combined incidence rates of EHS and HE at a shorter summer road race (11.3 km) reported an incidence rate of 3.10 runners per 1,000 finishers (Hosokawa et al., 2018). EHI incidence rates in mass-participation marathon events could range from 11.7 per 1,000 entrants in cold to mild environments (Ta = −4~16°C) (Roberts, 2000) to 148.9 per 1,000 entrants in hot environments (T_a_ = 32–34°C) (Ogwumike and Adeniyi, 2013). In elite-level international athletics championships, the incidence rate of EHI in marathon and race walking increases to 71.8 and 68.6 runners per 1,000 registered athletes, respectively (Hollander et al., 2021). However, the data observed in Sapporo exceeded further, where the average EHI incident rate in men's and women's marathons combined was 114.1 per 1,000 finishers, and the average EHI incidence rate of the three-race walks in Sapporo was 72.4 per 1,000 finishers. When examined by event, long-duration events that were held in the morning (men's 50 km race walk, men's marathon, and women's marathon) reported extremely high EHI incidence rates per 1,000 finishers, compared to shorter-distance events that were held in the late afternoon (men's and women's 20 km race walk). This suggests that the plan to avoid mid-day heat for long-duration events did not sufficiently work to reduce the risk of EHI in Sapporo, whose historical environmental condition is much milder than in Tokyo. While it may not be a conventional time schedule, race medicine in future elite endurance events that are being scheduled during the warm season may need to consider much earlier start times in the morning.

### EHI Cooling Rates

Five athletes (two EHS and three HE) were cooled using whole-body cold water immersion at the heat deck. The initial temperature of these athletes ranged from 39.55 to 42.6°C (Figure 3) with an average cooling rate of 0.11 ± 0.03°C to reach 39°C. This cooling rate is lower than the previously reported average cooling rate from 274 EHS runners who were treated at the Falmouth Road Race (average cooling rate, 0.22 ± 0.11°C) (Demartini et al., 2015). The reason for the slower cooling rate observed in our dataset compared to the Falmouth Road Race is unknown (for example, the heat deck in Sapporo was air-conditioned while the heat deck at the Falmouth Road Race is not); however, the difference in the total number of patients (Sapporo, *n* = 5; Falmouth Road Race, *n* = 274) and inter-variability of the body temperature response to whole-body cold water immersion may have influenced this results (Poirier et al., 2018; Hosokawa et al., 2020). Nevertheless, the cooling rate documented during marathons and race walks during the Games was sufficient to reduce the runner with the highest initial rectal temperature below 39°C within 30 min (Runner 11, Figure 3A), which is within the best medical practice of EHS prehospital management (Hosokawa et al., 2021). Therefore, our dataset suggests that the standardized EHS prehospital management implemented by the trained medical volunteers during the Tokyo Summer Olympic Games sufficiently addressed the medical emergency.

## Conclusion

In conclusion, most athletes (96%) who were admitted to the athlete medical station during the summer Olympic race walk and marathon had EHI. The incidence rate of EHI reported was higher than previously reported data from other races. Future organizers of elite endurance events (e.g., Paris 2024 Olympics, Los Angeles 2028 Olympics) are advised to consider much earlier start times (i.e., before sunrise) and to not only rely on historical data to identify the low-risk venue for EHI, but also have a contingency plan for extreme heat anomalies. In addition, standardized EHS prehospital management as outlined by Hosokawa et al. (2021) should be implemented in these events to optimize the treatment of EHS and severe HE.

## Data Availability Statement

The original contributions presented in the study are included in the article/supplementary material, further inquiries can be directed to the corresponding author.

## Author Contributions

MS created the main conceptual ideas for the article. All authors conducted a thorough review of the existing literature and contributed to the manuscript writing and review process. All authors contributed to the article and approved the submitted version.

## Conflict of Interest

The authors declare that the research was conducted in the absence of any commercial or financial relationships that could be construed as a potential conflict of interest.

## Publisher's Note

All claims expressed in this article are solely those of the authors and do not necessarily represent those of their affiliated organizations, or those of the publisher, the editors and the reviewers. Any product that may be evaluated in this article, or claim that may be made by its manufacturer, is not guaranteed or endorsed by the publisher.
